# Binuclear Cu(II) and Co(II) Complexes of Tridentate Heterocyclic Shiff Base Derived from Salicylaldehyde with 4-Aminoantipyrine

**DOI:** 10.1155/2012/601879

**Published:** 2012-05-07

**Authors:** Omar Hamad Shihab Al-Obaidi

**Affiliations:** Chemistry Department, College of Education for Women, Al-Anbar University, Ramadi, Iraq

## Abstract

New binuclear Co(II) and Co(II) complexes of ONO tridentate heterocyclic Schiff base derived from 4-aminoantipyrine with salicylaldehyde have been synthesized and characterized on the bases of elemental analysis, UV-Vis., FT-IR, and also by aid of molar conductivity measurements, magnetic measurements, and melting points. It has been found that the Schiff bases with Cu(II) or Co(II) ion forming binuclear complexes on (1 : 1) “metal : ligand” stoichiometry. The molar conductance measurements of the complexes in DMSO correspond to be nonelectrolytic nature for all prepared complexes. Distorted octahedral environment is suggested for metal complexes. A theoretical treatment of the formation of complexes in the gas phase was studied, and this was done by using the HyperChem-6 program for the molecular mechanics and semi-empirical calculations. The free ligand and its complexes have been tested for their antibacterial activities against two types of human pathogenic bacteria: the first type (*Staphylococcus aureus*) is Gram positive *and* the second type (*Escherichia coli*) is Gram negative (by using agar well diffusion method). Finally, it was found that compounds show different activity of inhibition on growth of the bacteria.

## 1. Introduction

Amino heterocyclic compounds containing two or more potential donor centers play an important role in the study of competitive reactivity of an bidentate ligand system [1]. Heterocyclic phenazone and their derivatives (4-aminoantipyrine) are known to act as bidentate or tridentate ligands when coordinated to metal ion [2]. 

Phenazone ligand can form mononuclear and binuclear complexes [3–5].

 Transition metal complexes containing a salicylaldehyde are commonly found in biological media and play important roles in processes such as catalysis of drug interaction with biomolecules [6]. Phenazone Schiff base chemistry is less extensive, and our laboratory has been exploring this chemistry [7–10].

**Figure 1:**
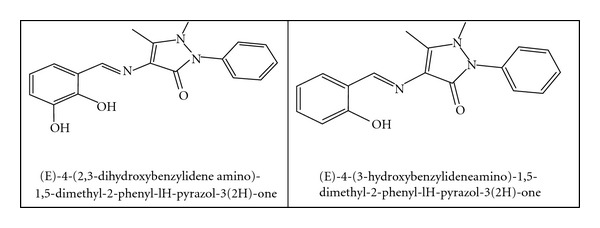
Structure of the ligands L1 and L2.

In this paper we are reporting the synthesis of the binuclear Co(II) and Cu(II) complexes of some heterocyclic Schiff base ligands (Figure 1) containing 4-aminoantipyrine. Spectral and magnetic studies have been used to characterize the structure of the complexes.

## 2. Experimental

### 2.1. Physical Measurements

A Fisher-100 infrared spectrophotometer was used to record the IR spectra as KBr and CsI disc, and UV/VIS spectra were measured by a HITACHI U-2000 spectrophotometer. Determination of all metals percentage by atomic absorption spectrophotometry on AA-680G (Shimadzu). Electrical conductance was measured on conductivity CDC304 (Jenway4070) melting points determined by an electric heated block apparatus (GallenKamp) and were uncorrected. Room temperature magnetic susceptibility measurements were carried out on a B.M 6 BRUKER type magnets, balance. Diamagnetic correction was done using pascal constants.

### 2.2. Materials

All the chemicals and solvents used for the synthesis were reagent grade and procured from (BDH chemicals or Sigma-Aldrich or Fluka). Metal salts were purchased from E. Merck and used as received. All solvents were dried and purified before used.

#### 2.2.1. Preparation of the Schiff Base Ligands (L1 and L2)

The ligands were prepared by condensation of 4-aminoantipyrine with salicylaldehyde in ethanol. This preparation was performed as cited in the literature [11].

The general structures of ligands obtained from chemical analysis and spectral methods are given in Figure 1. The full name of the ligand will be replaced with (L1 and L2) for the rest of this paper.

#### 2.2.2. Preparation of the Binuclear Metal Complexes

1.00 mm of the ligands were dissolved in 30 mL of ethanol, and solution of 1.00 mm of the metal salts [CuCl_2_·4H_2_O (0.20 g)] in 15 mL ethanol was added dropwise with continuous stirring. The mixture was stirred further for 2-3 h. at 80°C. The precipitated solid was then filtered off, washed with diethylether, followed by cold ethanol, and dried under vacuum. The same method was applied for the preparation of [CoCl_2_·6H_2_O] complexes by using the corresponding (L1 or L2) working in the same conditions with their respective molar ratio.

The physical properties of prepared complexes are listed in Table 1. The molar ratio of the complexes was determined according to the methods [12].

#### 2.2.3. Study of Biological Activity for Ligands (L1 and L2) and Their Metal Complexes

 The biological activity of the ligands and their metal complexes were studied against two selected types of bacteria which included *Escherichia coli* that are gram negative (−ve) and *Staphylococcus aureus* that are gram positive (+ve) to be cultivated and as control for the disc sensitivity test [13]. This method involves the exposure of the zone of inhibition toward the diffusion of microorganism on agar plat. The plates were incubated for (24 hours), at 37°C, and the zone of inhibition of bacteria growth around the disc was observed.

## 3. Result and Discussion

The Schiff bases ligands are soluble in common organic solvents. But its metal complexes are generally soluble in DMF and DMSO. The elemental analytical data of the complexes reveal that the compounds have “metal : ligand” an ion stoichiometry ratio of 1 : 1; the analytical data and other spectral analysis are in good agreement with the proposed stoichiometry of the complexes. The colors, yields, melting points, IR, and electronic absorption spectral data of all the compounds are presented in Table 2. The molar conductance of solutions of all the complexes in DMSO are in the range [5–8, 14] Ω^−1^ cm^2^ mole^−1^ Table 1. These observations suggest that all the complexes are nonelectrolytes [15] in DMSO (1∗10^−3^ M) at room temperature. Polydentate complexes were obtained from 1 : 1 molar ratio reactions with metal ions and L1 and L2 ligands. The ligands L1 and L2 on reaction with Cu(II) and Co(II) salt yields complexes are corresponding to the formulas [Cu_2_(L1)_2_H_2_O], [Co_2_(L1)_2_H_2_O], [Cu_2_(L2)_2_(H_2_O)_4_], and [Co_2_(L2)_2_(H_2_O)_4_].

### 3.1. Infrared Spectral Study

The most important infrared spectral bands of the investigated metal complexes in the present article are summarized in Table 2. The free Schiff base ligands are characterized by strong band at 1690, 1625, and 1270 cm^−1^ for L1, 1720, 1625, and 1290 cm^−1^ for L2 which may be ascribed to the stretching vibrations of C=O groups, C=N (imine) and C–O (phenolic) groups, respectively [7–10]. The band at 1630–1620 cm^−1^ due to the stretching mode of the C=N group in the spectrum of the free ligands shows a remarkable negative shift with splitting in the 1590–1610 cm^−1^ region in all the complexes spectra suggesting that the coordinating azomethine nitrogen atoms of the Schiff bases are involved in the complexes formation [7–10].

In the spectra of all binuclear complexes, the phenolic band at 1260–1270 cm^−1^ is shifted to lower frequency (10–30 cm^−1^). It is suggested that the oxygen atom of this phenolic (C–O) group is bridged to the metal ions. An additional band at 1160 cm^−1^ suggests that water molecules are coordinated to metal ion [16, 17]. This band may be assigned to water molecule OH heterocyclic ring vibration at 1580–1200 cm^−1^. Other band of M–O and M–N bands appear respectively at 535–515, 460–470 cm^−1^ Table 2.

### 3.2. Electronic Spectra and Magnetic Measurements

The electronic spectra were recorded in DMSO. In the spectrum of the ligand, the bands in the 380–340 nm range are assigned to the *n*-π* transitions of the azomethine group. During the formation of the complexes, these bands are shifted to lower wavelength, suggesting that the nitrogen atom of the azomethine group is coordinated to the metal ion. The values in the 325–245 nm range are attributed to the *π*-π* transition of the aromatic rings. In the spectra of the complexes, these bands are shifted slightly to lower wavelength.

### 3.3. Cu(II) Complexes

On the basis of the magnetic moment measurements, the Cu(II) complexes at room temperature probably have a binuclear structure with phenolic oxygen bridges. The magnetic moment lies in the range 0.9–1.69 B.M (for per Cu^+2^) Table 1, and this is abnormally small consistent with a dimeric structure [18, 19].

The electronic spectra of the copper complexes Table 1 recorded in DMSO supported a near octahedral geometry for them and support the proposal that H_2_O groups are coordinated axially to Cu (II) ions [10]. The spectrum of the Cu(II) complexes exhibits absorption bands at 11085, 16595, and 27990 cm^−1^.

These bands may be considered to the following three spin allowed [20] transitions:  ^2^B_1_g → ^2^A_1_g  (*dx* ^2^ − *y* ^2^ → *dz* ^2^),^2^B_1_g → ^2^B_2_g  (*dx* ^2^ − *y* ^2^ → *dz* *y*), and ^2^B → ^2^Eg  (*dx* ^2^ − *y* ^2^ → *dx* *y*, *dyz*), and these transitions suggest D_4h_ symmetry. The energy level sequence will depend on the amount of tetragonal distortion due to ligand field and Jahn Tellar distortion effect.

### 3.4. Co(II) Complexes

At room temperature the magnetic moment measurements of Co(II) complexes at 4.90–5.04 B.M correspond to three unpaired electrons, Table 1. 

The electronic spectra of all the Co(II) complexes display absorption at 10370, 14380, 18720, and 356960 cm^−1^; these bands may be assigned to the following transitions: ^4^T_1_g  (F) → ^4^T_2_g  (*ν*_1_), ^4^T_1_g → ^4^A_2_g  (*ν*_2_), and ^4^T_1_g(F) → ^4^T_2_g  (P)  (*ν*_3_), respectively [21]. It is difficult to give the assignments for the fourth band, and it may be due to chargetransfer. The position of electronic spectral bands indicates that these complexes have distorted octahedral geometry [17, 21, 22].

### 3.5. The Proposed Structure

**Figure 2:**
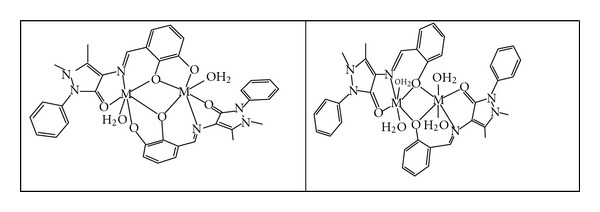
The proposed structure of the complexes where M = Cu(II) or Co(II)of the ligands L1 and L2.

According to the results obtained from (ir, uv/vis, molar ratio, molar conductivity, and atomic absorption) measurements for the prepared complexes, the proposed molecular structure of the complexes has an octahedral structure as shown in Figure 2.

### 3.6. Theoretical Study

**Figure 3:**
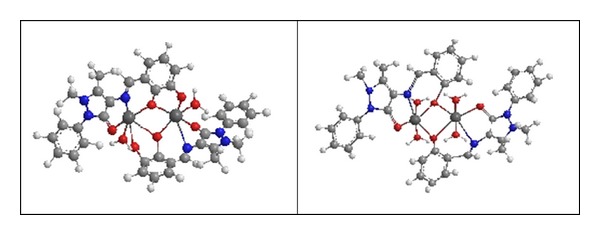
The optimized structural geometry of Cu(II) complexes.

The ball and cylinders and some of selected structural parameters (bond length and angles) of the optimized geometries are shown in Figure 3 and Table 3.

As shown in this figure, there is no obvious trend for the variation of these parameters. The values of the bond length and angles of the optimized geometries are quite similar to the experimental results of the corresponding compounds.

### 3.7. Antibacterial Studied

**Figure 4:**
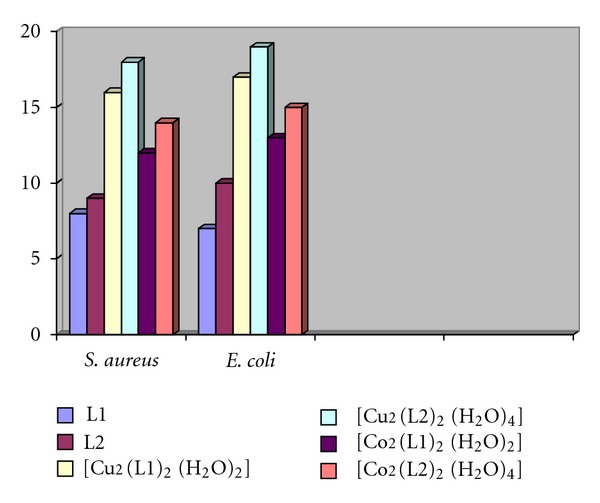
The effect of ligands and their metal complexes toward bacteria.

The ligand and its transition metal complexes were evaluated against different species of bacteria [23, 24]. The antibacterial action of the ligands and the complexes of Co(II) and Cu(II) was checked by the disc diffusion technique. This was done on *Staphylococcus aureus* (gram positive) and *Escherichia coli* (gram negative) bacteria at 25°C. The disc of whatman no. 4 filter paper having the diameter 6.00 mm was soaked in the solution of compounds in DMSO (1.0 mg cm^−1^). After drying it was placed on nutrient agar plates. The inhibition area wasobserved after 48 hr. DMSO used as control Figure 4. The moderate effect was observed with Cu(II) complexes against *Staphylococcus aureus*, which is known as a resistant to most commercial antibiotic. The Schiff base ligands had more antibacterial activity than other ligands used, and this effect may be due to the presence of –ph, –OH and –N=C– groups which are electron releasing. The antibacterial results evidently showed that the activity of the ligand compounds became more pronounced when coordination to the metal ions.

## Figures and Tables

**Table 1:** Analytical and physical data of all the complexes.

No.	Complexes/FW	Colour	ΔM (Ω^−1^ cm^2^ mol^−1^) In DMSO	M.P (°C)	Yield (%)	Elemental analysis				
						(% found) % Cal.				
						C	H	M	N	O
1	[Cu_2_(L1)_2_(H_2_O)_2_] C_36_H_34_Cu_2_N_6_O_8_/805	Light orange	9	>240	75	53.66 (53.61)	4.25 (4.21)	15.77 (15.73)	10.43 (10.39)	15.88 (15.84)
2	[Cu_2_(L2)_2_(H_2_O)_4_] C_36_H_40_Cu_2_N_6_O_8_/811	Orange	7	>240	65	53.26 (53.21)	4.97 (4.93)	15.65 (15.60)	10.35 (10.30)	15.77 (15.73)
3	[Co_2_(L1)_2_(H_2_O)_2_] C_36_H_34_Co_2_N_6_O_8_/796	Light brown	5	>240	70	54.28 (54.23)	4.30 (4.26)	16.07 (16.02)	14.80 (14.76)	10.55 (10.50)
4	[Co_2_(L2)_2_(H_2_O)_4_] C_36_H_40_Co_2_N_6_O_8_/802	Brown	8	>240	75	53.87 (53.83)	5.02 (4.98)	14.69 (14.65)	10.47 (10.41)	15.95 (15.90)

**Table 2:** Characteristic IR and electronic spectral data of the metal complexes.

No.	Complexes	UV/ VIS *λ* max (cm^−1^)	*μ*_ eff._ BM	IR spectra cm^−1^				
				*υ*C=O	*υ*C=N	*υ*C–O	*υ*M–N	*υ*M–O
1	[Cu_2_(L1)_2 _H_2_O]	11085, 16595, 27990	0.9	1650 s	1600 m	1265 s	460 w	530 w
2	[Cu_2_(L2)_2_(H_2_O)_4_]	11080, 16590, 27995	1.69	1670 s	1610 m	1260 s	465 w	535 w
3	[Co_2_(L1)_2_H_2_O]	10370, 14380, 18720, 35690	5.04	1665 s	1610 m	1270 s	470 w	520 w
4	[Co_2_(L2)_2_(H_2_O)_4_]	10375, 14385, 18725, 35695	4.90	1660 s	1590 m	1270 s	460 w	515 w

**Table 3:** Structural parameters, bond length (°A), and angles (°) of the [Cu_2_(L2)_2_(H_2_O)_4_]complex.

	Parameters		
Bond lengths (°A)		Bond angles (°)	
Cu(136)–O(138) Cu(135)–O(137) O(134)–Cu(135) O(129)–Cu(135) O(129)–Cu(136) N(125)–Cu(136) O(116)–Cu(136) O(110)–Cu(136) O(105)–Cu(136) O(105)–Cu(135) N(101)–Cu(135) O(92)–Cu(135) Cu(50)–O(52) Cu(49)–O(51) O(48)–Cu(49) C(47)–O(48) C(46)–H(82) C(46)–C(47) C(45)–H(81) C(45)–C(46) C(44)–H(80) C(44)–C(45) O(43)–Cu(49) O(43)–Cu(50) O(30)–Cu(50) C(29)–C(37) C(28)–N(39) C(28)–C(29) C(27)–O(30) C(27)–C(28) N(26)–C(31) N(26)–C(27) N(25)–C(38)	1.5230 1.5230 2.0400 2.3621 1.9129 2.3774 1.8092 1.3403 1.8419 1.7708 1.3875 1.5551 1.5228 1.5227 2.0398 1.4033 1.1000 1.0149 1.1000 1.1328 1.1000 1.0148 2.3617 1.9130 1.8090 1.0468 1.0144 1.1317 1.1265 0.9997 0.9735 1.0636 0.9597	O(19)–Cu(50)–O(52)–H(85) O(19)–Cu(50)–O(52)–H(86) O(24)–Cu(50)–O(52)–H(85) O(24)–Cu(50)–O(52)–H(86) O(30)–Cu(50)–O(52)–H(85) O(30)–Cu(50)–O(52)–H(86) N(39)–Cu(50)–O(52)–H(85) N(39)–Cu(50)–O(52)–H(86) O(43)–Cu(50)–O(52)–H(85) O(43)–Cu(50)–O(52)–H(86) O(6)–Cu(49)–O(51)–H(83) O(6)–Cu(49)–O(51)–H(84) N(15)–Cu(49)–O(51)–H(83) N(15)–Cu(49)–O(51)–H(84) O(19)–Cu(49)–O(51)–H(83) O(19)–Cu(49)–O(51)–H(84) O(43)–Cu(49)–O(51)–H(83) O(43)–Cu(49)–O(51)–H(84) O(48)–Cu(49)–O(51)–H(83) O(48)–Cu(49)–O(51)–H(84) C(47)–O(48)–Cu(49)–O(6) C(47)–O(48)–Cu(49)–N(15) C(47)–O(48)–Cu(49)–O(19) C(47)–O(48)–Cu(49)–O(43) C(47)–O(48)–Cu(49)–O(51) Cu(50)–O(43)–Cu(49)–O(48) Cu(50)–O(43)–Cu(49)–O(51) C(42)–O(43)–Cu(50)–O(19) C(42)–O(43)–Cu(50)–O(24) C(42)–O(43)–Cu(50)–O(30) C(42)–O(43)–Cu(50)–N(39) C(42)–O(43)–Cu(50)–O(52) Cu(49)–O(43)–Cu(50)–O(19) Cu(49)–O(43)–Cu(50)–O(24) Cu(49)–O(43)–Cu(50)–O(30)	179.9998 −0.0001 174.1519 −5.8480 −40.8405 139.1596 9.7808 −170.2191 −3.4183 176.5818 −179.9999 0.0011 −122.7109 57.2900 41.5484 −138.4507 37.5946 −142.4044 37.5946 142.4044 −155.1509 −34.7220 55.8245 52.8179 −127.1821 180.000 180.000 −150.107 −143.209 66.7882 18.0306 32.7885 −2.8955 4.0021 −146.00
